# MicroRNA-222 reprogrammed cancer-associated fibroblasts enhance growth and metastasis of breast cancer

**DOI:** 10.1038/s41416-019-0566-7

**Published:** 2019-09-04

**Authors:** Annesha Chatterjee, Samir Jana, Soumya Chatterjee, Laura M Wastall, Gunjan Mandal, Nelofar Nargis, Himansu Roy, Thomas A Hughes, Arindam Bhattacharyya

**Affiliations:** 1 Immunology Laboratory, Department of Zoology, University of Calcutta, Kolkata, West Bengal India; 2 Department of Cellular Pathology, St James’s University Hospital, Leeds, UK; 3 Department of Surgery, Medical College, Kolkata, West Bengal India; 4 School of Medicine, University of Leeds, Leeds, UK

**Keywords:** Breast cancer, Cancer microenvironment, Metastasis, Cell migration, Senescence

## Abstract

**Background:**

Cancer-associated fibroblasts (CAFs) are known to impact on tumour behaviour, but the mechanisms controlling this are poorly understood.

**Methods:**

Breast normal fibroblasts (NFs) or CAFs were isolated from cancers by laser microdissection or were cultured. Fibroblasts were transfected to manipulate miR-222 or Lamin B receptor (LBR). The fibroblast-conditioned medium was collected and used to treat epithelial BC lines MDA-MB-231 and MDA-MB-157. Migration, invasion, proliferation or senescence was assessed using transwell, MTT or X-gal assays, respectively.

**Results:**

MiR-222 was upregulated in CAFs as compared with NFs. Ectopic miR-222 expression in NFs induced CAF-like expression profiles, while miR-222 knockdown in CAFs inhibited CAF phenotypes. LBR was identified as a direct miR-222 target, and was functionally relevant since LBR knockdown phenocopied miR-222 overexpression and LBR overexpression phenocopied miR-222 knockdown. MiR-222 overexpression, or LBR knockdown, was sufficient to induce NFs to show the CAF characteristics of enhanced migration, invasion and senescence, and furthermore, the conditioned medium from these fibroblasts induced increased BC cell migration and invasion. The reverse manipulations in CAFs inhibited these behaviours in fibroblasts, and inhibited paracrine influences on BC cells.

**Conclusion:**

MiR-222/LBR have key roles in controlling pro-progression influences of CAFs in BC. This pathway may present therapeutic opportunities to inhibit CAF-induced cancer progression.

## Background

Breast cancer (BC) is the leading cause of cancer death worldwide among women,^1^ and nearly 2.3 million females are newly diagnosed annually.^2^ Although there are initial responses to treatment, many cancers relapse and distant metastases occur in nearly one-third of woman; these are typically fatal.^3^ The biology behind BC metastases still remains undetermined. Therefore, understanding molecular determinants of metastasis is crucial for finding new therapeutic strategies. The tumour microenvironment consists of immune cells, blood vessels, endothelial cells, fibroblasts and extracellular matrix.^4^ This microenvironment plays key roles in disease outcome by inducing tumour cell proliferation and aggressiveness.^5,6^ Cancer-associated fibroblasts (CAFs), an activated form of tissue-resident fibroblasts present within breast cancers, comprise a major component of the tumour microenvironment,^7^ characterised most commonly by expression of α-smooth muscle actin.^8^ CAFs can induce cancer progression^9^ and metastasis^10^ by secreting various cytokines, chemokines and growth factors (e.g., VEGF, FGF2, TGFβ, CXCL12, IL6 and IL8)^11,12^ and by modulating the extracellular matrix (ECM) that facilitates tumour cell migration and invasion.^13,14^ CAFs also modulate immune cell function to create an immune-suppressive environment during cancer progression.^15^ The process of transformation of CAFs from resident normal fibroblasts is achieved by several growth factors,^16–18^ however, the mechanisms of transformation have not yet been fully explored and may represent attractive targets for therapeutic intervention; interestingly, once transformed into CAFs, the CAF phenotype has been regarded as stable, probably though maintenance of epigenetic changes.^19^

MicroRNAs (miRNAs) are small, non-coding RNAs that regulate gene expression by binding to the 3′untranslated regions (3′UTRs) of target genes, leading to post-transcriptional downregulation.^20^ MiRNAs can be oncogenic or act as tumour suppressors, depending upon the specific genes that they regulate.^21,22^ Dysregulation of miRNAs within the fibroblast component of breast cancer has been reported, and is therefore implicated in induction or maintenance of the CAF phenotype. For example, differential expression between breast CAFs and normal fibroblasts has been reported for many miRNAs, including miR-221, miR-31, miR-205, miR-200b, miR-200c, miR-107, miR-30b, the let-7 family and miR-26b,^23,24^ although there is relatively little consistency between reports. Far fewer miRNAs have been shown experimentally to have functional impacts on the breast CAF phenotype, or most interestingly indirectly on cancer cell behaviour; examples include miR-26b,^24^ let-7b,^25^ miR-200 family^26^ and miR-29b.^27^ Although some of these studies have indicated functional roles for specific miRNAs within CAFs, very few studies have directly investigated miRNA roles on the transformation and maintenance of CAF phenotypes and on how this impacts on cancer behaviour, therefore how miRNAs in CAFs are involved in cancer progression remains poorly understood. Here, we have focussed on miR-222 as a candidate regulator of CAF phenotypes and cancer behaviour. MiR-222 has previously been reported as an oncogenic miRNA in various cancers, including BC,^28–32^ functioning within the cancer cells themselves, as opposed to stromal cells. Furthermore, it has been shown that upregulation of miR-222 induced growth of cancer cells by targeting p27/kip1,^32^ as well as chemoresistance by targeting PTEN/Akt.^33^ Interestingly, in the context of our study on function in fibroblasts, levels of miR-222 positively correlated with fibroblast viability in hypertrophic scar tissues,^34^ while miR-222 has been shown to induce replicative senescence in human lung fibroblasts.^35^ However, to date, no studies have been published on roles of miR-222 in CAFs. Therefore, the above observations suggest the importance of miR-222 as a post-transcriptional modifier, playing functional roles in BC progression. However, it is unclear whether expression of miR-222 is only tumour cell specific or the other cells also express miR-222 in tumour microenvironment. As miR-222 is an important oncogenic factor in breast cancer, we wanted to unveil whether CAFs also expressed miR-222 to influence disease progression.

## Materials and methods

### Reagents

Foetal bovine serum (FBS), DMEM and antibiotic/antimycotic (100X; 10,000 units/mL penicillin, 10,000 µg/mL streptomycin, 25 µg/mL amphotericin B) were purchased from ThermoFisher (MA, USA). Antibodies for α-smooth muscle actin (ab5694), LBR (ab32535) and β-actin (ab8227) were purchased from Abcam (MA, USA), and for vimentin (#5741S) and Slug (#9585S) from Cell Signaling Technology (MA, USA). Secondary anti-rabbit antibody was purchased from Bangalore Genei (Bangalore, India). AlexaFluor 488-conjugated anti-rabbit and TRITC-conjugated anti-rabbit antibodies were purchased from ThermoFisher (MA, USA). MiR-222-3p mimics (#MSY0000279) and inhibitors (#MIN0000279), miScript inhibitor negative control (#1027271), siLBR (#SI00035798) and AllStars negative control siRNA (#1027280) were purchased from Qiagen (Hilden, Germany). Lipofectamine 3000 was purchased from ThermoFisher (MA, USA). β-galactosidase assay kit was purchased from Cell Signaling Technology (MA, USA). KpnI, XbaI, PmeI, NotI and T4 DNA ligase were purchased from New England Biolabs (MA, USA).

### Ethical issues and cell culture

Ethical approval for collection and use of human tissue was obtained from the Leeds East REC (references 06/Q1206/180, 09/H1306/108), and also from the Ethical Committee, Medical College, Kolkata, references MC/KOL/IEC/NON-SPON/102/09-2015. Fresh samples of surgically removed breast cancer samples were minced, digested with collagenase IV from HiMedia Laboratories Pvt. Ltd (Mumbai, India) and plated (collagenase I coated) to derive primary breast CAFs (from within tumour masses) and NFs (from > 1 cm outside tumour margins). Breast cancer cell lines MDA-MB-231 and MDA-MB-157 were obtained from the ATCC (VA, USA). The NF and CAF fibroblast lines were derived from breast cancer samples and immortalised using lentiviral transduction of hTERT, as previously described.^24^ All cell lines were cultured in the DMEM with 10% FBS and 1% antibiotics/antimycotics in a humid atmosphere incubator with 5% CO_2_ at 37 °C. NFs were transfected with scrambled siRNA negative control (NC), siLBR, miR-222 mimic or mimic control using Lipofectamine 3000 (ThermoFisher; MA, USA) according to the manufacturer’s instruction. Similarly, CAFs were transfected with scramble negative control, miR-222 inhibitor, pCDNA or pCDNA-LBR. Seventy-two hours after transfection, cells were harvested for western blotting, qRT-PCR analyses, senescence studies or migration/invasion studies. To collect NF and CAF conditioned medium, cells were seeded into six-well plates at 1.5 × 10^5^ cells/well. As previously described, cells were transfected as appropriate for 24 h, then medium was replaced with fresh DMEM. After 48 and 72 h of incubation, the medium was collected and centrifuged at 150 *g* for 5 min, and then the supernatant was collected.

### RNA isolation and qPCR

**Fig. 1:**
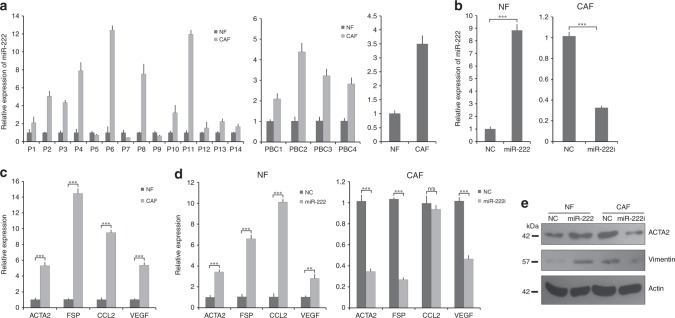
MiR-222 is upregulated in breast cancer-associated fibroblasts (CAFs), and controls breast fibroblast phenotype. **a** MiR-222 expression was determined by qPCR, and is shown for CAFs relative to NFs in three separate cell types. Left plot: matched pairs of normal fibroblasts (NFs) and CAFs were isolated from breast cancer patient samples using laser microdissection of archival (fixed) tissue. The data represent technical triplicates. Middle plot: four matched pairs of primary cultured CAFs and NFs from breast cancer patient-derived tumour samples. The data represent technical triplicates. Right plot: immortalised breast CAF and NF cell lines. The data represent biological triplicates. **b** Immortalised breast NFs (left) or CAFs (right) were transfected with miR-222 mimics or control (NC), or miR-222 inhibitor (i) or control (NC) and miR-222 expression was assessed using qPCR. Data represent biological triplicates. **c** Relative gene expression levels of the CAF markers, α-SMA, Fibroblast Specific Protein (FSP), CCL2 and VEGF were assessed in immortalised CAFs as compared with immortalised NFs using qPCR. The data represent biological triplicates. **d** Relative expression levels of the same CAF markers were assessed using qPCR in NFs (left) or CAFs (right) transfected with miR-222 mimics or control (NC), or miR-222 inhibitor (i) or control (NC). The data represent biological triplicates. **e** NFs or CAFs were transfected with miR-222 mimics or control (NC), or miR-222 inhibitor (i) or control (NC) and protein expression of the CAF markers α-SMA (ACTA2) and vimentin were assessed using Western blots, along with β-actin as a loading control. ***p < 0.0005 and **p < 0.005

For LMD samples, the total RNA was extracted from using RecoverAll Total Nucleic Acid Isolation Kit for FFPE (ThermoFisher, MA, USA) following the manufacturer’s protocols, or the mirVanaTM miRNA Isolation Kit (ThermoFisher, MA, USA). RNA was reverse-transcribed for miR-222 using the TaqMan MicroRNA RT kit (ThermoFisher, MA, USA), and the resulting first strand was amplified using specific Taqman miRNA assay primers (ThermoFisher, MA, USA). The PCRs were performed by StepOne plus detection system (ThermoFisher, MA, USA), and amplification data were normalised using RNU6 expression. Relative expression levels were calculated using the 2^–ΔΔCt^ method. For cultured primary fibroblasts (in Fig. 1b), RNA was isolated from a 90-mm dish of cultured cells using TRIzol (ThermoFisher, MA, USA), according to the manufacturer's instruction and RNA quantified by MULTISCANGO (ThermoFisher, MA, USA). For miRNA expression status and gene expression status, first strand cDNA and cDNA were synthesised with miScript RT-II kit (Qiagen, Hilden, Germany) and Superscript III (ThermoFisher, MA, USA), respectively. MiR-222 expression was analysed using PCR starter kits (Qiagen, Hilden, Germany); U6 was used as an endogenous control. Quantitative RT-PCR was performed using the Power SYBR Green Master Mix (ThermoFisher, MA, USA) on StepOne detection system (ThermoFisher, MA, USA). Expression was normalised to the house-keeping gene 18S.

### Protein extraction and western blots

Cells were washed with PBS and lysed in RIPA lysis buffer System (lysis buffer pH 7.4, 200 mM PMSF, protease inhibitor cocktail, 100 mM sodium orthovanadate) (Santa Cruz-sc24948). The lysates were centrifuged at 12700 g for 20 min at 4 ˚C. Protein concentrations were determined using BCA Protein Assay Kit (ThermoFisher, MA, USA). Total protein was separated by SDS-PAGE (12% gel) then transferred to the PVDF membranes (Millipore) and blocked with 5% skimmed milk. The membrane was then incubated overnight with primary antibodies. Protein bands were detected by incubation with horseradish peroxidase (HRP)-conjugated antibodies (Bangalore Genei, Bangalore, India). Bands were visualised using ECL.

### Histology, laser capture microdissection (LMD) and immunohistochemistry (IHC)

LMD was carried out on the Zeiss PALM Laser Capture Microdissection Microscope exactly as described previously.^24^ In brief, archival FFPE cancer blocks and matched normal blocks were obtained and sectioned at 10 μm onto Membrane Slides NF 1.0 PEN (Zeiss, Oberkochen, Germany). A guide section was stained with haematoxylin and eosin and reviewed by a histopathologist (LMW) to identify areas of fibroblasts with very few admixed inflammatory cells, epithelial cells or necrosis. The equivalent areas were then identified on other sections, and these were collected with the laser into lids of AdhesiveCap500 opaque PCR Tubes (Zeiss, Oberkochen, Germany) by laser pressure catapulting (LPC). The microscope settings used for LCM were cut energy 71, focus 65, LPC energy 100, focus 65 at ×100 magnification. Areas dissected from each case varied between 5.2 and 27.4×10^6^ μm^2^ depending on fibroblast density. For IHC, FFPE human breast cancer resection tissue was available; cancerous and non-cancerous breast tissue were immunostained for α-SMA and LBR. The signal was amplified and visualised with 3, 3-diaminobenzidine (DAB) chromogen, followed by counterstaining with haematoxylin.

### Luciferase reporter assays

**Fig. 2:**
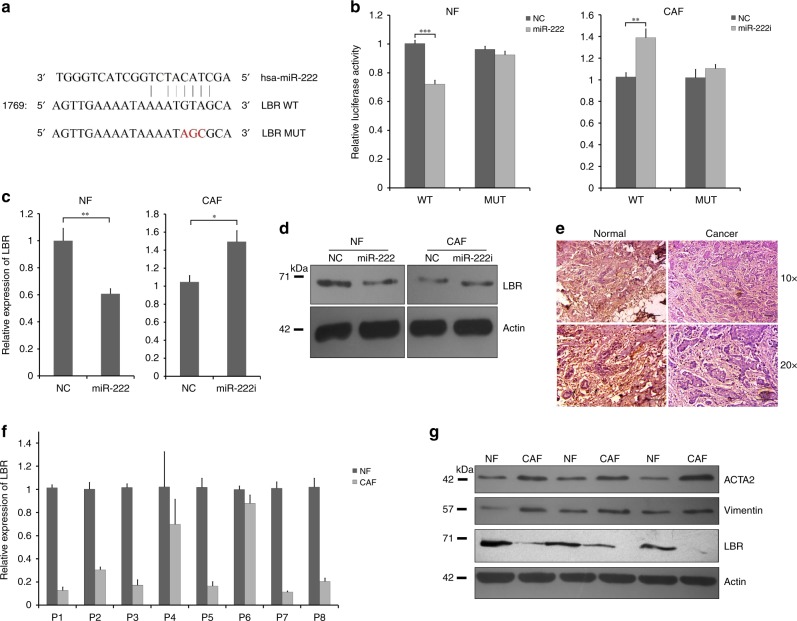
Lamin B Receptor (LBR) is a direct target of miR-222 in breast fibroblasts and is downregulated in breast CAFs relative to matched NFs. **a** LBR was identified as a potential miR-222 target by bioinformatics. The potential miR-222-binding site within the LBR 3′UTR is shown (WT), along with a binding-dead mutant used experimentally (MUT). **b** Luciferase reporters were cloned that allow expression of luciferase transcripts containing the wild-type or mutant LBR miR-222 3′UTR binding sites. These were transfected into NFs (left) or CAFs (right) along with either miR-222 mimic or control (NC), or miR-222 inhibitor (i) or control (NC), and relative luciferase activity was determined. The data represent two biological replicates. **c**, **d** NFs or CAFs were transfected as shown, and the relative expression of endogenous LBR was determined using qPCR (**c**) or western blots (**d**). qPCR data represent three biological replicates. **e** Representative matched pair of normal and breast cancer tissues was assessed for expression of LBR by immunohistochemistry (×10 and ×20 magnification). Brown staining represents expression of the target protein, while pink is a counterstain. **f** Relative expression of LBR was assessed in eight pairs of primary cultured NFs and CAFs isolated from breast cancer patient-derived tumour. The data represent technical triplicates. **g** Protein expression levels of LBR, and the CAF markers α-SMA and vimentin were assessed by western blotting in three pairs of primary cultured NFs and CAFs isolated from breast cancer patient-derived tumour samples. β-actin was used as a loading control. ***p < 0.0005, **p < 0.005, *p < 0.05

The potential miR-222 binding sequence from the LBR gene (WT LBR) and three nucleotide-mutated LBR (MUT LBR) (Fig. 2a) were cloned into pMiRGLO (Promega, WI, USA) under restriction sites PmeI and XbaI (primers listed in Table S1). Luciferase activities were measured 48 h after transfection using Dual-Luciferase Reporter Assays (Promega, WI, USA).

### Cell proliferation assays

NFs and CAFs were seeded in 96-well plates at 1 × 10^4^ cells/well and transfected the following day. Cell proliferation was monitored after 72 h by MTT (HiMedia Laboratories, Mumbai, India). In total, 10 µl of MTT solution (10 mg/ml) in 100 µl of DMEM media was added per well. After 4 h of incubation, formazan complexes were dissolved in 100 µl of DMSO, and signals were measured using MULTISCANGO plate reader (ThermoFisher, MA, USA). To evaluate the effects of conditioned media, MDA-MB-231 and MDA-MB-157 were seeded in 96-well plates at 2 × 10^4^ cells/well. Twenty-four hours after seeding, condition media was applied. MTT assays were performed at 48 h as above.

### Cell migration and invasion assays

Cell migration and invasion ability were determined by Corning transwell insert chambers (8-µm pores; Corning, MA, USA) and Matrigel (Sigma Aldrich, MO, USA) coated in a transwell chamber for invasion analysis. Conditional media from different treatment conditions were used in lower chambers for migration and invasion study. The cells were prior treated with conditional media for 48 h. In total, 1 × 10^5^ cells of MDA-MB-231 or MDA-MB-157 were added into chambers and incubated for 28 h at 37 °C. Cells that had migrated or invaded were fixed with 100% methanol, stained with 0.5% crystal violet, imaged and counted manually.

### Senescence-associated beta-galactosidase (SA-β-gal) assays

SA-β-gal activity was detected by β-gal staining kit (Cell Signaling Technologies) according to the manufacturer's instructions. Cells containing blue stain were counted manually as positive senescent cells, and images were taken under phase contrast.

### Cell immunostaining and confocal microscopy

NFs and CAFs were allowed to attach to glass coverslips overnight at 37 ˚C. Cells were fixed in 4% paraformaldehyde (20 min) and permeabilised with 0.1% Triton X-100 (15 min). Blocking was performed with 5% BSA for 1 h followed by incubation with α-SMA and vimentin primary antibodies (4 ˚C overnight). AlexaFluor 488-conjugated and TRITC-conjugated secondary antibodies were incubated for 1 h. Coverslips were counterstained with DAPI and analysed on a confocal microscope (Olympus).

### Cloning

For the LBR-overexpressing vector, LBR open-reading frame was cloned within pCDNA 3.1+ vector using KpnI and XbaI restriction sites and primers GTGGTACCACCATGCCAAGTAGGAAATTTG (forward) and GCTCTAGAGCTTAGTAGATGTATGGAAATATACGG (reverse).

### Statistical analyses

All data are representative of at least three independent experiments. The results are presented as means ±SEM of at least three independent experiments in all the figures. Differences were considered statistically significant at *p* < 0.05 using Student’s *t* test.

## Results

### miR-222 is upregulated in breast CAFs, and controls CAF phenotypes

Many studies have shown that miRNAs are dysregulated in CAFs. To investigate roles of miR-222 dysregulation in CAF biology, we first assessed relative expression of miR-222 in 14 matched pairs of normal fibroblasts (NFs) and cancer-associated fibroblasts (CAFs) that have been isolated from breast cancer resection samples using laser microdissection (LMD). We found miR-222 to be upregulated in CAFs relative to matched NFs in 11 out of 14 cases, representing significant upregulation overall by a mean fold of 3.63 (*p* < 0.04; Fig. 1a, left plot). To expand this finding, we further assessed miR-222 expression in four matched pairs of primary human breast NFs and CAFs cultures extracted from breast cancer resections; we found a greater than twofold induction of miR-222 in CAFs as compared with matched NFs in all cases (Fig. 1a, middle plot). Finally, miR-222 expression in immortalised human breast NFs was compared with immortalised human breast CAFs; this also showed higher expression of miR-222 in CAFs by more than threefold (Fig. 1a, right plot). We concluded that miR-222 is more highly expressed in CAFs than NFs in breast tissue.

To explore functional roles of miR-222 in transformation of NFs to CAFs, we conducted a series of experiments on immortalised human breast NFs or immortalised human breast CAFs using miR-222 mimics to upregulate expression in NFs, or miR-222 inhibitors to downregulate expression in CAFs. First, miR-222 expression was assessed using qRT-PCR to confirm appropriate over- or under-expression (Fig. 1b); miR-222 was significantly overexpressed by more than eightfold and was knocked down to one-third of its original level. Next, the relatively “normal” or CAF-like phenotypes of these cell lines were characterised to establish their baselines for subsequent analyses. Expression of a range of classical CAF markers^7,8,12^ was assessed using qRT-PCR (Fig. 1c), immunofluorescence or western blotting (Fig. S1). Relative expressions of ACTA2 (smooth muscle actin), FSP, CCL2 and VEGF were all substantially higher in CAFs as compared with NFs (a minimum of fivefold higher as assessed by qRT-PCR), as indicative of the CAF phenotype. Upregulation in CAFs of *ACTA2*/smooth muscle actin and vimentin was also confirmed at the protein level (Fig. S1). Next, NFs were transfected with miR-222 mimics or control mimics, and CAFs were transfected with miR-222 inhibitors or control inhibitors, and miR-222′s influence on transformation of NFs to CAFs, or on maintenance of the CAF phenotype was examined using the same panel of CAF markers as previously. Transfection of miR-222 mimics in NFs resulted in significant upregulation of all the CAF markers (qRT-PCR, Fig. 1d, left plot; western blots, Fig. 1e). Furthermore, downregulation of miR-222 in CAFs using miR-222 inhibitors significantly downregulated expression of the CAF markers, with the notable and surprising exception of CCL2 (qRT-PCR, Fig. 1d, right plot; western blots, Fig. 1e). We concluded that miR-222 levels play key roles in controlling the CAF phenotype in breast fibroblasts.

### LBR is a direct target of miR-222

To investigate potential mechanisms by which miR-222 plays roles in breast fibroblast biology, bioinformatics tools were used to predict potential target genes of miR-222. We analysed the best possible miR-222 seed matches using miRanda, TargetScan and miRDB software. Eleven predicted target genes were chosen for further analysis based on the target scores, containing seed matches for miR-222. On analysis of expression by qRT-PCR in NFs and paired CAFs from patient samples three of these genes, RECK, THOP1 and LBR, demonstrated differential expression in the predicted direction. LBR showed the most substantial and consistent dysregulated result in NFs and CAFs, and therefore LBR was prioritised for subsequent analyses. The potential miR-222 binding site within the LBR 3′UTR is depicted in Fig. 2a. We performed a number of different assays to assess whether LBR is a true direct target of miR-222 within breast fibroblasts. First, luciferase reporter constructs containing either the wild-type miR-222 binding site from the LBR 3′UTR or a mutated (non-binding) version of the site (Fig. 2a) were cloned. Reporters were co-transfected with miR-222 mimics and inhibitors, or appropriate controls, in NFs and CAFs respectively, and luciferase assays were performed. MiR-222 mimics significantly inhibited the activity of the luciferase reporter containing the wild-type LBR site, while the mutated site was insensitive to miR-222 overexpression in NFs (Fig. 2b, left plot), whereas miR-222 inhibitors significantly increased the activity of the wild-type reporter, while the mutated reporter was again insensitive in CAFs (Fig. 2b, right plot). Next, endogenous LBR expression was assessed in NFs after transfection to overexpress miR-222, and in CAFs after transfection with miR-222 inhibitors (Fig. 2c, d). LBR expression was downregulated by miR-222 overexpression and upregulated by miR-222 inhibition at the level of both transcript (Fig. 2c) and protein (Fig. 2d), findings that were in accordance with it being downstream of miR-222 function. We concluded that LBR is a direct target of miR-222 in breast fibroblasts, via a canonical miR-222 binding site within the LBR 3′UTR.

### Downregulation of LBR induces a CAF-like phenotype

Our next aim was to determine whether LBR downregulation is involved in determining the breast CAF phenotype, since we have already determined that CAFs overexpress miR-222 and that this can downregulate LBR. In one approach, we examined whether breast normal stroma or tumour stroma exhibit differential expression of LBR using immunohistochemistry. LBR expression (Fig. 2e) was relatively reduced in tumour stroma, in contrast to ACTA2 (smooth muscle actin) (Fig. S2), which was relatively high in tumour stroma. In addition, qRT-PCR was performed on eight matched pairs of primary NF and CAF cultures. LBR expression was significantly reduced by a mean of 4.3-fold in CAFs compared with the NFs (*p* < 0.01), in contrast to expression of the CAF markers ACTA2 and vimentin, which were upregulated in CAFs as expected and indicative of the CAF phenotype (Fig. 2f, g).

**Fig. 3:**
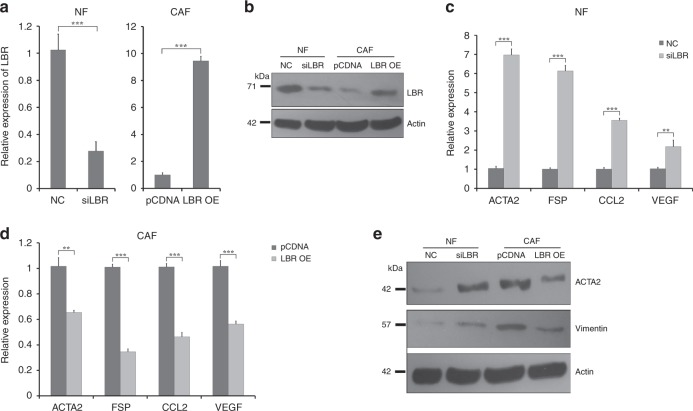
LBR regulates the NF vs CAF breast fibroblast phenotype. **a**, **b** Immortalised breast NFs (left) or CAFs (right) were transfected with siRNA targeting LBR (siLBR) or control (NC), or plasmid to allow overexpression of LBR (LBR OE) or control plasmid (pCDNA), and LBR expression was assessed using qPCR (**a**) or western blots (**b**). qPCR data represent biological triplicates, while β-actin represents a loading control for the western analysis. **c**–**e** Immortalised breast NFs (**c**, **e**) or CAFs (**d**, **e**) were transfected as shown, and the relative expression levels of the CAF marker genes α-SMA, fibroblast-specific protein (FSP), CCL2 and VEGF were determined by qPCR (**c**, **d**), or of α-SMA and vimentin by western blot (**e**). qPCR data represent biological triplicates, while β-actin represents a loading control for the western analysis. ***p < 0.0005 and **p < 0.005

We next assessed whether LBR downregulation was sufficient for transformation of NFs to CAFs, and conversely whether LBR overexpression would reduce CAF features. We transfected immortalised breast NFs with siRNA targeted against LBR or with an appropriate non-targeting control, and also transfected immortalised breast CAFs with plasmids to allow overexpression of LBR, or with appropriate control plasmids. The efficacy of knockdown and overexpression was confirmed using qRT-PCR (Fig. 3a) and western blots (Fig. 3b); knockdown was to ~30% of the original levels, while overexpression was by more than ninefold. Next, we assessed expression of our panel of CAF markers in these cells after knockdown or overexpression of LBR using qRT-PCR or western blots as previously. Reduction of LBR expression in NFs, and increased expression of LBR in CAFs, significantly increased and decreased, respectively, the expression of all the CAF markers (Fig. 3c–e). We concluded that LBR is a key regulator of the breast CAF phenotype, and that knockdown of LBR alone is sufficient to transform NFs into cells that resemble CAFs.

### MiR-222 and LBR regulate migration, invasion and senescence in breast fibroblasts

**Fig. 4:**
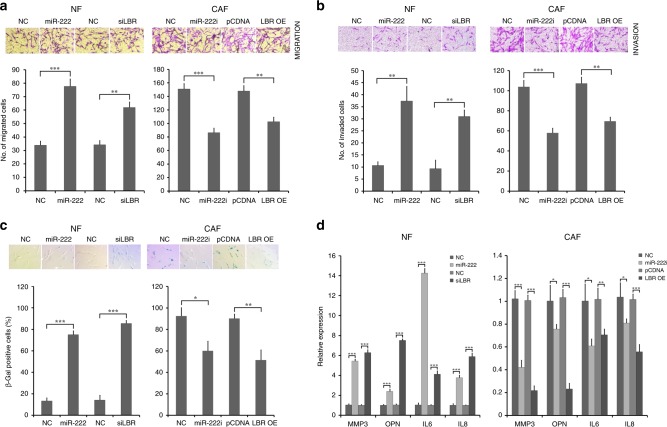
MiR-222 and its downstream target LBR modulate the behaviour of breast fibroblasts. Immortalised breast NFs were transfected with miR-222 mimic or siRNA targeting LBR (siLBR) or appropriate controls (NC), and immortalised breast CAFs were transfected with miR-222 inhibitor (i) or to overexpress LBR (LBR OE) or with appropriate controls (NC, pCDNA). **a**, **b** Migration (**a**) or invasion (**b**) of fibroblasts was assessed using transwell assays. Representative images are shown (**a**, **b**, upper panels), along with quantified data that represent biological triplicates (**a**, **b**, lower panels). **c** Expression of senescence-associated β-galactosidase was also assessed by X-gal staining and is shown as blue green colouration (upper panels). Positive cells were quantified in data that represent biological triplicates (lower panels). **d** Expression of the senescence markers MMP3 and osteopontin (OPN) and senescence-associated secretory phenotype markers IL6 and IL8 were assessed by qPCR in NFs (left plot) and CAFs (right plot). The data represent biological triplicates. ***p < 0.0005, **p < 0.005, *p < 0.05

Since it has been previously demonstrated that CAFs are characterised by higher cell motility than their adjacent NFs,^36^ we also investigated whether miR-222 overexpression or siRNA-mediated knockdown of LBR would induce increased NF migration or invasion, and conversely whether miR-222 inhibition or LBR overexpression would reduce CAF migration or invasion. Immortalised breast NFs or CAFs were transfected as previously with miR-222 mimics or inhibitors, respectively, or with siRNA targeted against LBR or overexpression of LBR, respectively. We then performed migration or invasion assays using transwell assays. NFs transfected with miR-222 mimics or siRNA targeting LBR had significantly higher migration and invasion capacity (Fig. 4a, b, left plots), whereas inhibition of miR-222 or LBR overexpression in CAFs significantly reduced their migration and invasion capacity (Fig. 4a, b, right plots). In order to confirm that these apparent effects on migration and invasion did not relate to differences in cell numbers induced by the transfections, we also evaluated proliferation capabilities of fibroblasts after these manipulations of miR-222 or LBR expression. We performed MTT assays after the same transfected conditions as mentioned above; there were no significant differences in proliferation of NFs or CAFs after any of these treatments (Fig. S3). We concluded that miR-222 and its target LBR have key influences on aspects of CAF-like fibroblast behaviour, namely migration and invasion.

It was previously reported that loss of LBR is associated with induction of cellular senescence,^37^ therefore, we also specifically evaluated senescence by determining senescence-associated β-galactosidase (SA-β-gal) activities in NFs and CAFs after dysregulation of expression of miR-222 or LBR. We found an increased SA-β-gal activity in NFs transfected with miR-222 mimics or siLBR compared to controls (Fig. 4c, left plot). In addition, inhibition of miR-222 activity or increased LBR expression in CAFs significantly reduced SA-β-gal activity (Fig. 4c, right plot). We also further examined features of this senescence phenotype by determining whether it was also associated with differential expression of other senescence markers. NFs and CAFs were transfected as before, and qRT-PCR was used to assess expression of MMP3, OPN, IL6 and IL8, which are upregulated in senescent cells particularly those showing the senescence-associated secretory phenotype (SASP).^38,39^ Expression of all four markers was significantly upregulated in NFs transfected with miR-222 mimic or siLBR (Fig. 4d, left plot), while expression was significantly downregulated in CAFs transfected with miR-222 inhibitors or to overexpress LBR (Fig. 4d, right plot). This provided strong support that changes in β-gal activity we had detected were indeed associated with senescence, and that miR-222 and LBR expression impact on senescence phenotypes in breast fibroblasts.

### miR-222 and LBR in breast fibroblasts regulate proliferation, migration and invasion of cancer cells

**Fig. 5:**
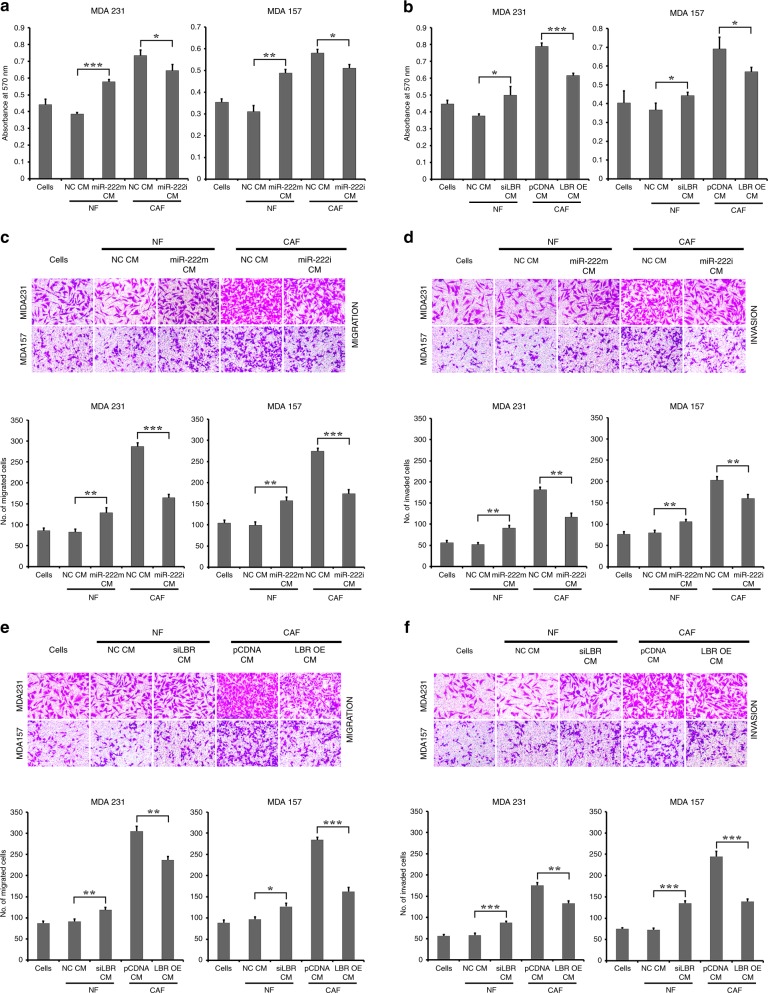
MiR-222 and LBR control the ability of breast fibroblasts to influence cancer cell proliferation and metastatic potential. Immortalised breast NFs were transfected with miR-222 mimic or siRNA targeting LBR (siLBR) or appropriate controls (NC), and immortalised breast CAFs were transfected with miR-222 inhibitor (i) or to overexpress LBR (LBR OE) or with appropriate controls (NC, pCDNA). Conditioned medium (CM) was collected from fibroblast cultures and used to treat breast epithelial cancer lines MDA-MB-231 or MDA-MB-157. **a**, **b** Proliferation of MDA-MB-231 or MDA-MB-157 cell lines cultured with conditioned medium (CM) from the transfected fibroblasts as labelled was determined using MTT assay. The data represent biological triplicates. **c**–**f** Migration (**c**, **e**) or invasion (**d**, **f**) of epithelial cancer cells was assessed using transwell assays. Representative images are shown (upper panels), along with quantified data that represent biological triplicates (lower panels). ***p < 0.0005, **p < 0.005, *p < 0.05

To better understand the exact effects of miR-222 and its action on LBR in fibroblasts on epithelial breast cancer cell behaviour, we assessed abilities of NFs and CAFs after manipulation of miR-222 or LBR expression to induce growth or migration/invasion of breast cancer cells. In particular, we focused on the highly metastatic MDA-MB-231 and the relatively less metastatic and triple-negative MDA-MB-157 cell lines. In order to assess influences of fibroblasts on the cancer cells, we decided to collect conditioned medium from fibroblasts and treat cancer cells with this. Fibroblast-conditioned medium has frequently been shown to influence cancer cell behaviour in this context.^36^ NFs or CAFs were transfected exactly as previously. Conditional medium (CM) was collected from these cells after transfection, and was added to cultures of epithelial breast cancer cells. First, proliferation of epithelial cells was assessed using MTT assays after treatment with CM (Fig. 5a, b). In both epithelial cell lines, CM from control NFs caused a marginal, but not significant, reduction in proliferation, while CM from control CAFs caused a marked and significant increase in proliferation when compared with cells without CM (*p* < 0.005), as has been reported previously.^36^ More interestingly, overexpression of miR-222 in NFs and knockdown of miR-222 in CAFs significantly altered the activity of these CMs on both epithelial cell lines; miR-222 overexpression in NFs produced CM that had similar growth inducing effects to that from control CAFs, while knockdown of miR-222 in CAFs reduced this growth stimulation (Fig. 5a). Changes in LBR expression within NFs and CAFs also had similar significant effects, with LBR knockdown allowing CM from NFs to induce relative growth increases, while overexpression of LBR in CAFs reduced the growth stimulatory effect of CAF CM (Fig. 5b). We concluded that upregulation of miR-222 and downregulation of LBR were both necessary and sufficient for CAF function with respect to inducing proliferation of breast cancer cells. Next, we investigated the influences of these CMs on the motility of BC cells. BC cells also responded dramatically and significantly to these CMs in terms of migration and invasion capacities. CM from NFs had little influence on migration, while CM from CAFs significant and consistently induced migration (Fig. 5c, e) and invasion (Fig. 5d, f) in both epithelial cell lines. Overexpression of miR-222 or knockdown of LBR in NFs led to enhanced migration in the epithelial lines. Knockdown of miR-222 or overexpression of LBR in CAFs led to reduced migration in the epithelial lines. Exactly the same result was evident in terms of invasion, with miR-222 and LBR having a key influence on abilities of NFs or CAFs to induce invasion in both epithelial cell lines (Fig. 5c–f). To further clarify that differences in migration/invasion of the breast cancer epithelial cells treated with CM did not solely relate to changes in proliferation rate, we additionally performed an experiment assessing proliferation in the cells treated exactly as for the migration/invasion assay (Fig. S4), which involves an earlier time point than that used for Fig. 5a, b. There were no significant differences in almost all combinations, supporting the conclusion that true differences in migration/invasion were seen. An exception is MDA-MB-157 cells treated with CAF CM, in which a significant reduction in proliferation was seen with CM after miR-222 knockdown and LBR overexpression; however, this change in proliferation was only up to 20% (Fig. S4), whereas decreases in migration/invasion were up to 50% (Fig. 5e, f), indicating that—again—true influences on migration/invasion were seen.

**Fig. 6:**
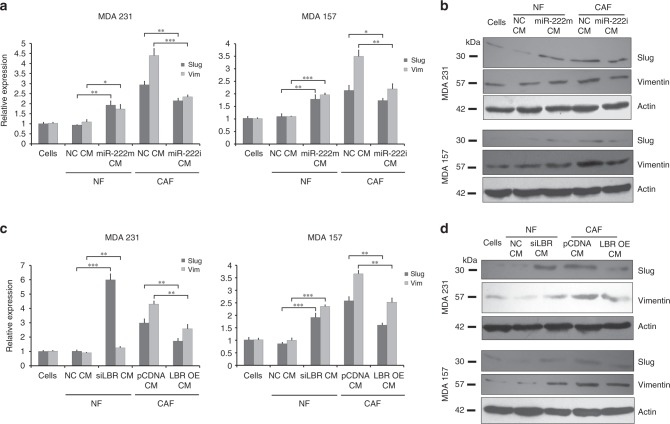
MiR-222 and LBR control the ability of breast fibroblasts to activate EMT in breast cancer cells. Immortalised breast NFs were transfected with miR-222 mimic or siRNA targeting LBR (siLBR) or appropriate controls (NC), and immortalised breast CAFs were transfected with miR-222 inhibitor (i) or to overexpress LBR (LBR OE) or with appropriate controls (NC, pCDNA). Conditioned medium (CM) was collected from fibroblast cultures and used to treat breast epithelial cancer lines MDA-MB-231 or MDA-MB-157. Expression levels of the EMT-associated genes slug and vimentin were assessed in MDA-MB-231 or MDA-MB-157 cell lines cultured with the conditioned medium (CM) from the transfected fibroblasts as labelled using qPCR (**a**, **c**) or western blots (**b**, **d**). qPCR data represent biological triplicates, while β-actin represents a loading control for the western analysis. ***p < 0.0005, **p < 0.005, *p < 0.05

Finally, to determine whether the fibroblasts were influencing migration and invasion capacity of epithelial cells by inducing epithelial to mesenchymal transition (EMT) pathways, we analysed expression of EMT markers slug and vimentin in the BC cells after culture with the same CMs, using both qRT-PCR and western blots (Fig. 6). CM from NFs had little influence on slug or vimentin expression at either transcript or protein level, while CM from CAFs induced up to a sixfold increase in expression. Manipulation of miR-222 or LBR expression in the fibroblasts consistently and significantly altered slug and vimentin expression in accordance with the changes in migration and invasion, with greater slug and vimentin expression in cells induced to migrate/invade more by miR-222 mimics or LBR siRNA in NFs, and reduced slug and vimentin expression in cells induced to migrate/invade less by miR-222 inhibition or LBR overexpression in CAFs. We concluded that miR-222 and, downstream of this, LBR have key influences on abilities of breast fibroblasts to control critical cancer-related behaviours of breast epithelial cancer cells, including proliferation, migration, invasion and EMT.

## Discussion

Increasing evidence supports the proposal that miRNA dysregulation within CAFs modulates function of the tumour microenvironment. However, it is worth highlighting that most studies have emphasised investigation of miRNA roles within the tumour cells themselves, therefore it remains the case that relatively little is known about roles of miRNAs in the tumour microenvironment. In this study, we have focussed on roles of miR-222 within fibroblasts.

We initially compared the miR-222 expression difference between CAFs and adjacent NFs in breast cancer tissue samples. Our study demonstrated that miR-222 was significantly upregulated in CAFs. Although others have made similar comparisons for multiple miRNAs in this context,^23,24,26,40^ miR-222 has not previously been identified as differentially expressed. This may be because studies have not always effectively purified only the fibroblasts for their analyses, as we have by LMD, or may be because miR-222 was not always included in the analyses. Next, we identified that miR-222 targets Lamin B receptor (LBR) expression in the CAFs. Various targets of miR-222 have been published previously, but LBR is a novel target on which we focused since we were able to confirm its downregulation in CAFs in accordance with negative regulation by the upregulated miR-222. Most importantly, downregulation of LBR with siRNA phenocopied miR-222 overexpression in functional experiments using breast fibroblasts, strongly suggesting that LBR is a key functionally important target of miR-222 in these cells.

In terms of cellular function, we showed that miR-222 and LBR were both involved in defining and maintaining the breast CAF phenotype in terms of expression patterns, and fibroblast behaviours including migration and invasion. MiR-222 has previously been assigned roles in promotion of cell proliferation, migration, invasion and drug resistance within cancer cells,^32,33,41^ so our findings are in accordance with this, although they represent the first observations to our knowledge in a cancer stromal cell type. With respect to functions of LBR, a role in senescence has previously been reported in fibroblasts^37^ and we also observed a related result with manipulation of LBR expression by siRNA, overexpression or via miR-222 all impacting on senescence in breast fibroblasts. LBR encodes the lamin B receptor, a 70.4-kD protein of the inner nuclear membrane,^42^ which is known to interact with heterochromatin and B-type lamins.^43^ Other functions for the protein have been implied by observations that ectopic LBR expression deregulates differentiation of olfactory neurons inducing an un- or early-differentiated state.^44^

More excitingly, we showed that fibroblasts with differential expression of miR-222 or LBR have potent differential influences on behaviour of two independent breast epithelial cancer cell lines. We demonstrated that conditioned medium from fibroblasts that were stimulated to be more CAF-like by miR-222 overexpression or siLBR promoted breast cancer proliferation, migration and invasion. Similarly, abilities of conditioned medium from CAFs to promote breast cancer proliferation, migration and invasion was inhibited by reduced miR-222 levels or by LBR overexpression. Next, we investigated possible pathways involved in the breast tumour motility effects. We found that the enhanced migration and invasion were associated with increased expression of vimentin and slug in the BC cells, suggesting involvement of the EMT pathway. Taken together, these results suggest that miR-222 upregulation, and subsequent LBR downregulation, within fibroblasts in the tumour microenvironment contribute to progression of BC, through induction of proliferation and EMT-related motility. It is established that cancer progression is not only dependent on tumour cells themselves but also dependent on the tumour microenvironment^45,46^ and, in particular, CAFs, which promote growth and invasion of cancer cells through synthesis and remodelling of ECM and secreting some growth factors.^4,47^ Hence, blocking CAF activity may be a key approach to effectively control cancer metastasis. Manipulation of either miR-222 expression or LBR function provide candidate approaches for development of these types of anti-cancer therapies. In particular, our data demonstrate that reduced expression of miR-222 or overexpression of LBR in CAFs greatly reduces their ability to promote aggressive behaviours in cancer cells, supporting the proposal that CAF phenotypes are not fixed and could be normalised therapeutically.

Surprisingly, our data also implicate the miR-222/LBR axis in senescence induction in CAFs. Previous studies have indicated that increased miR-222 or loss of LBR induces senescence,^35,48^ so this is not a surprise in itself; however, it is unexpected that high levels of senescence in CAFs would be associated with cancer cell behaviours that promote progression, since senescence is often regarded as a tumour-suppressor mechanism in cancer cells.^49^ However, more recent evidence shows that senescent cells may promote oncogenesis through secretion of secretory factors (SASP)^38^ and several reports describe abilities of senescent human fibroblasts to promote growth and tumorigenesis.^39^ In support of this, here, we demonstrated that miR-222 overexpression in NFs induces a SASP phenotype, leading to increased secretion of at least the SASP factors IL6 and IL8, and the classical senescence marker MMP3.

In conclusion, we present miR-222 and LBR as key molecules involved in transformation and maintenance of breast CAFs, which in turn therefore impacts on the aggressive tumorigenic behaviour of breast cancer cells. These molecules are novel targets for therapeutic intervention.

## Supplementary information

Supplementary Material

## Data Availability

Data and material shall be available from the corresponding authors.

## References

[1] Ürun Y, Utkan G, Yalcin B, Akbulut H, Onur H, Oztuna DG (2015). The role of cardiac biomarkers as predictors of trastuzumab cardiotoxicity in patients with breast cancer. Exp Oncol.

[2] Dharmica April Haridatt Mistry and Peter William French. (2016). Circulating phospholipids as biomarkers of breast cancer: a review. Breast Cancer.

[3] Redig AJ1, McAllister SS (2013). Breast cancer as a systemic disease: a view of metastasis. J. Intern. Med..

[4] Liotta LA, Kohn EC (2001). The microenvironment of the tumour-host interface. Nature.

[5] Hanahan D, Coussens LM (2012). Accessories to the crime: functions of cells recruited to the tumor microenvironment. Cancer Cell.

[6] Fang H, Declerck YA (2013). Targeting the tumor microenvironment: from understanding pathways to effective clinical trials. Cancer Res..

[7] Kalluri R (2016). The biology and function of fibroblasts in cancer. Nat. Rev. Cancer.

[8] Serini G, Gabbiani G (1999). Mechanisms of myofibroblast activity and phenotypic modulation. Exp. Cell Res..

[9] Li Y, Wang S, Ni HM, Huang H, Ding WX (2014). Autophagy in alcohol-induced multiorgan injury: mechanisms and potential therapeutic targets. Biomed. Res. Int..

[10] Kadera BE, Li L, Toste PA, Wu N, Adams C, Dawson DW (2013). MicroRNA-21 in pancreatic ductal adenocarcinoma tumor-associated fibroblasts promotes metastasis. PLos One.

[11] De Palma M, Biziato D, Petrova TV (2017). Microenvironmental regulation of tumour angiogenesis. Nat. Rev. Cancer.

[12] Kalluri R, Zeisberg M (2006). Fibroblasts in cancer. Nat. Rev. Cancer.

[13] Erez N, Truitt M, Olson P, Arron ST, Hanahan D (2010). Cancer-associated fibroblasts are activated in incipient neoplasia to orchestrate tumor-promoting inflammation in an NF-κB-dependent manner. Cancer Cell.

[14] Ostman A, Augsten M (2009). Cancer-associated fibroblasts and tumor growth–bystanders turning into key players. Curr. Opin. Genet. Dev..

[15] Givel AM, Kieffer Y, Scholer-Dahirel A, Sirven P, Cardon M, Pelon F (2018). miR200-regulated CXCL12β promotes fibroblast heterogeneity and immunosuppression in ovarian cancers. Nat. Commun..

[16] Brown LF, Guidi AJ, Schnitt SJ, Van De Water L, Iruela-Arispe ML, Yeo TK (1999). Vascular stroma formation in carcinoma in situ, invasive carcinoma, and metastatic carcinoma of the breast. Clin. Cancer Res..

[17] Elenbaas B, Weinberg RA (2001). Heterotypic signaling between epithelial tumor cells and fibroblasts in carcinoma formation. Exp. Cell Res..

[18] Strutz F, Zeisberg M, Hemmerlein B, Sattler B, Hummel K, Becker V (2000). Basic fibroblast growth factor expression is increased in human renal fibrogenesis and may mediate autocrine fibroblast proliferation. Kidney Int..

[19] DeClerck YA, Pienta KJ, Woodhouse EC, Singer DS, Mohla S (2017). The tumor microenvironment at a turning point knowledge gained over the last decade, and challenges and opportunities ahead: a white paper from the NCI TME network. Cancer Res.

[20] Bartel DP (2004). MicroRNAs: genomics, biogenesis, mechanism, and function. Cell.

[21] Guo J, Gong G, Zhang B (2018). miR-539 acts as a tumor suppressor by targeting epidermal growth factor receptor in breast cancer. Sci. Rep..

[22] Lv C, Li F, Li X, Tian Y, Zhang Y, Sheng X (2017). MiR-31 promotes mammary stem cell expansion and breast tumorigenesis by suppressing Wnt signaling antagonists. Nat. Commun..

[23] Zhao L, Sun Y, Hou Y, Peng Q, Wang L, Luo H (2012). MiRNA expression analysis of cancer-associated fibroblasts and normal fibroblasts in breast cancer. Int. J. Biochem. Cell Biol..

[24] Verghese ET, Drury R, Green CA, Holliday DL, Lu X, Nash C (2013). MiR-26b is down-regulated in carcinoma-associated fibroblasts from ER-positive breast cancers leading to enhanced cell migration and invasion. J. Pathol..

[25] Al-Harbi B, Hendrayani SF, Silva G, Aboussekhra A (2018). Let-7b inhibits cancer-promoting effects of breast cancer-associated fibroblasts through IL-8 repression. Oncotarget.

[26] Tang X, Hou Y, Yang G, Wang X, Tang S, Du YE (2016). Stromal miR-200s contribute to breast cancer cell invasion through CAF activation and ECM remodeling. Cell Death Differ.

[27] Liu Y, Zhang J, Sun X, Su Q, You C (2017). Down-regulation of miR-29b in carcinoma associated fibroblasts promotes cell growth and metastasis of breast cancer. Oncotarget.

[28] Yang X, Yang Y, Gan R, Zhao L, Li W, Zhou H (2014). Down-regulation of mir-221 and mir-222 restrain prostate cancer cell proliferation and migration that is partly mediated by activation of SIRT1. PLoS One.

[29] Zhong C, Ding S, Xu Y, Huang H (2015). MicroRNA-222 promotes human non-small cell lung cancer H460 growth by targeting p27. Int. J. Clin. Exp. Med..

[30] Quintavalle C, Garofalo M, Zanca C, Romano G, Iaboni M, del Basso De Caro M (2012). miR-221/222 overexpession in human glioblastoma increases invasiveness by targeting the protein phosphate PTPμ. Oncogene.

[31] Zhang DQ, Zhou CK, Jiang XW, Chen J, Shi BK (2014). Increased expression of miR-222 is associated with poor prognosis in bladder cancer. World J. Surg. Oncol..

[32] Sun C, Li N, Zhou B, Yang Z, Ding D, Weng D (2013). miR-222 is upregulated in epithelial ovarian cancer and promotes cell proliferation by downregulating P27kip1. Oncol Lett..

[33] Wang DD, Li J, Sha HH, Chen X, Yang SJ, Shen HY (2016). miR-222 confers the resistance of breast cancer cells to Adriamycin through suppression ofp27(kip1) expression. Gene.

[34] Zhang Y, Lin X, Zhang L, Hong W, Zeng K (2018). MicroRNA-222 regulates the viability of fibroblasts in hypertrophic scars via matrix metalloproteinase 1. Exp. Ther. Med..

[35] Markopoulos GS, Roupakia E, Tokamani M, Vartholomatos G, Tzavaras T, Hatziapostolou M (2017). Senescence-associated microRNAs target cell cycle regulatory genes in normal human lung fibroblasts. Exp. Gerontol..

[36] Baroni S, Romero-Cordoba S, Plantamura I, Dugo M, D'Ippolito E, Cataldo A (2016). Exosome-mediated delivery of miR-9 induces cancer-associated fibroblast-like properties in human breast fibroblasts. Cell Death Dis..

[37] Lukášová E, Kovarˇík A, Bacˇíková A, Falk M, Kozubek S (2017). Loss of lamin B receptor is necessary to induce cellular senescence. Biochem. J..

[38] Coppe JP, Desprez PY, Krtolica A, Campisi J (2010). The senescence-associated secretory phenotype: the dark side of tumor suppression. Annu Rev. Pathol..

[39] Lugo R, Gabasa M, Andriani F, Puig M, Facchinetti F, Ramírez J (2016). Heterotypic paracrine signaling drives fibroblast senescence and tumor progression of large cell carcinoma of the lung. Oncotarget.

[40] Du YE, Tu G, Yang G, Li G, Yang D, Lang L (2017). MiR-205/YAP1 in activated fibroblasts of breast tumor promotes VEGF-independent angiogenesis through STAT3 signaling. Theranostics.

[41] Liu W, Song N, Yao H, Zhao L, Liu H, Li G (2015). miR-221 and miR-222 simultaneously target RECK and regulate growth and invasion of gastric cancer cells. Med. Sci. Monit..

[42] Holmer L, Pezhman A, Worman HJ (1998). The human lamin B receptor/sterol reductase multigene family. Genomics.

[43] Duband-Goulet I, Courvalin JC, Buendia B (1998). LBR, a chromatin and lamin binding protein from the inner nuclear membrane, is proteolyzed at late stages of apoptosis. J. Cell Sci..

[44] Clowney EJ, LeGros MA, Mosley CP, Clowney FG, Markenskoff-Papadimitriou EC, Myllys M (2012). Nuclear aggregation of olfactory receptor genes governs their monogenic expression. Cell.

[45] Junttila MR, de Sauvage FJ (2013). Influence of tumour micro-environment heterogeneity on therapeutic response. Nature.

[46] Luo Z, Wang Q, Lau WB, Lau B, Xu L, Zhao L (2016). Tumor microenvironment: the culprit for ovarian cancer metastasis?. Cancer Lett.

[47] Martinez-Outschoorn UE, Lisanti MP, Sotgia F (2014). Catabolic cancer-associated fibroblasts transfer energy and biomass to anabolic cancer cells, fueling tumor growth. Semin. Cancer Biol..

[48] Gorospe M, Abdelmohsen K (2011). MicroRegulators come of age in senescence. Trends Genet..

[49] Serrano M, Lin AW, McCurrach ME, Beach D, Lowe SW (1997). Oncogenic ras provokes premature cell senescence associated with accumulation of p53 and p16INK4a. Cell.

