# Supplementary material for: MicroRNA-222 reprogrammed cancer-associated fibroblasts enhance growth and metastasis of breast cancer
Source: Br J Cancer. 2019 Sep 4;121(8):679–89. doi: 10.1038/s41416-019-0566-7 (PMC6889135; doi:10.1038/s41416-019-0566-7)
Supplement: Supplementary file 1 — Supplementary Material [file 41416_2019_566_MOESM1_ESM.pdf]

SUPPLEMENTARY FIGURES:

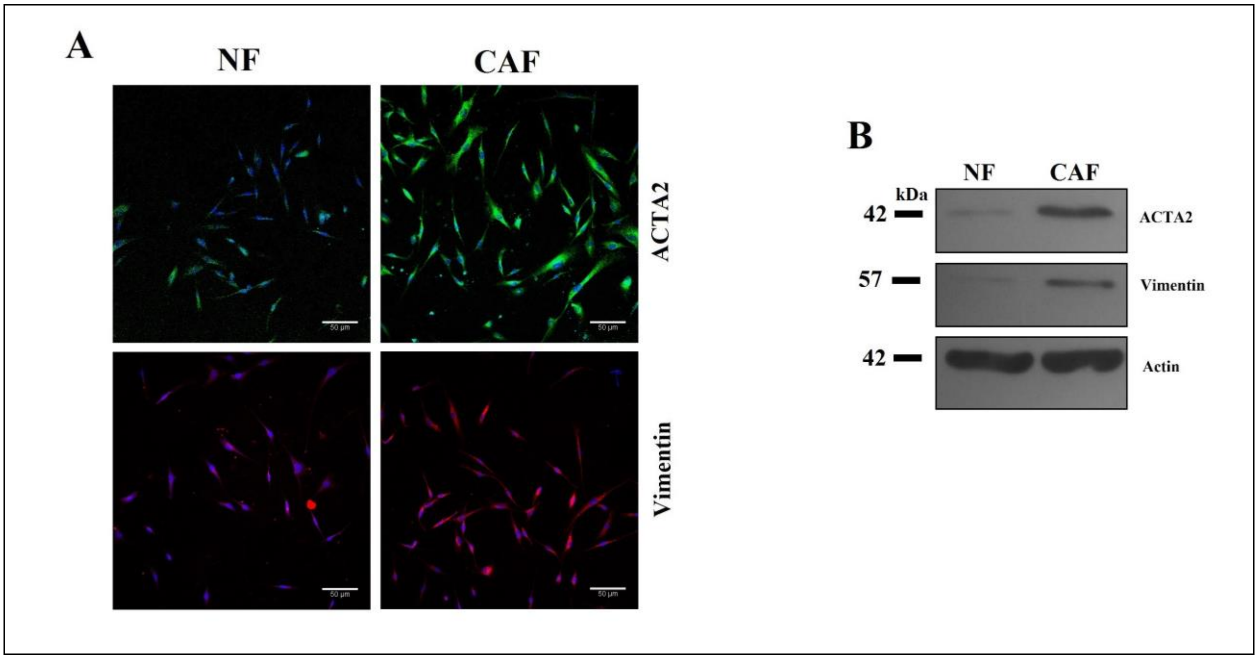

Supplementary Figure S1: Relative expression of the CAF marker, α-SMA / ACTA2 and Vimentin was assessed in immortalized breast NFs and CAFs by (A) immunofluorescence or (B) by Western blot (using β-Actin as a loading control).

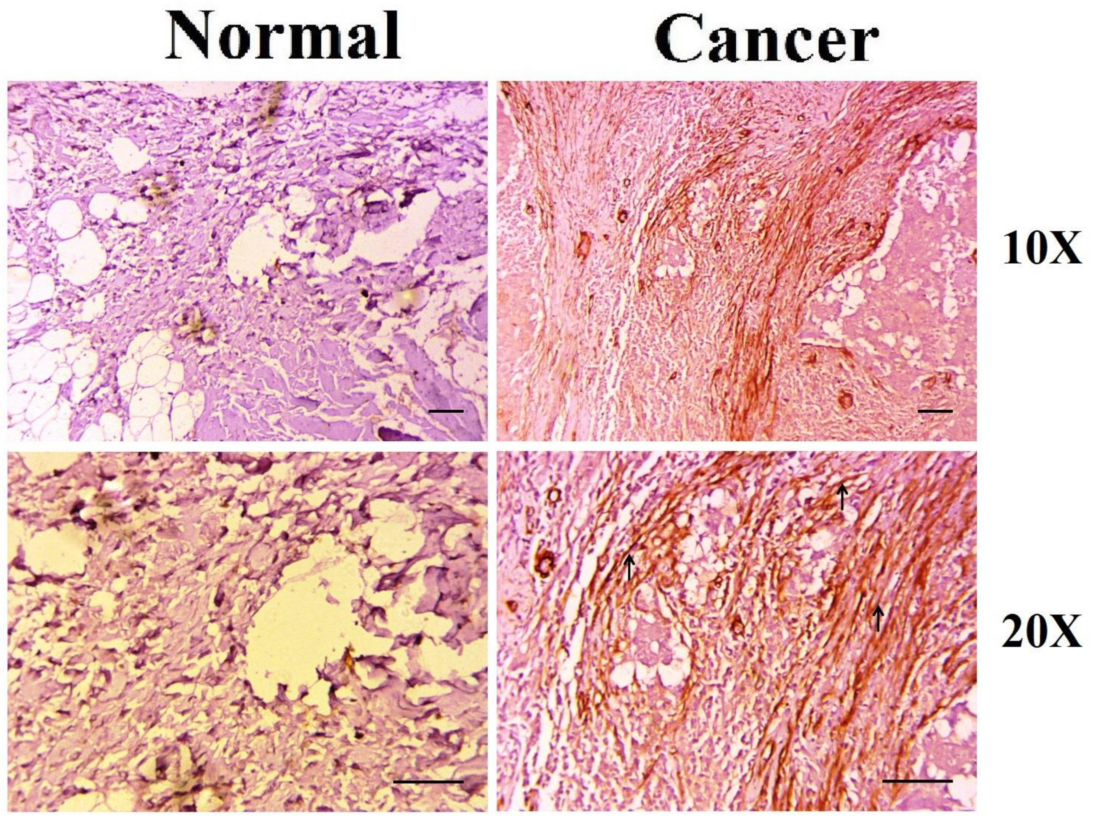

Supplementary Figure S2: Representative matched pair of normal and breast cancer tissues was assessed for expression of CAF marker α-Sma by immunohistochemistry (10X and 20X magnification).

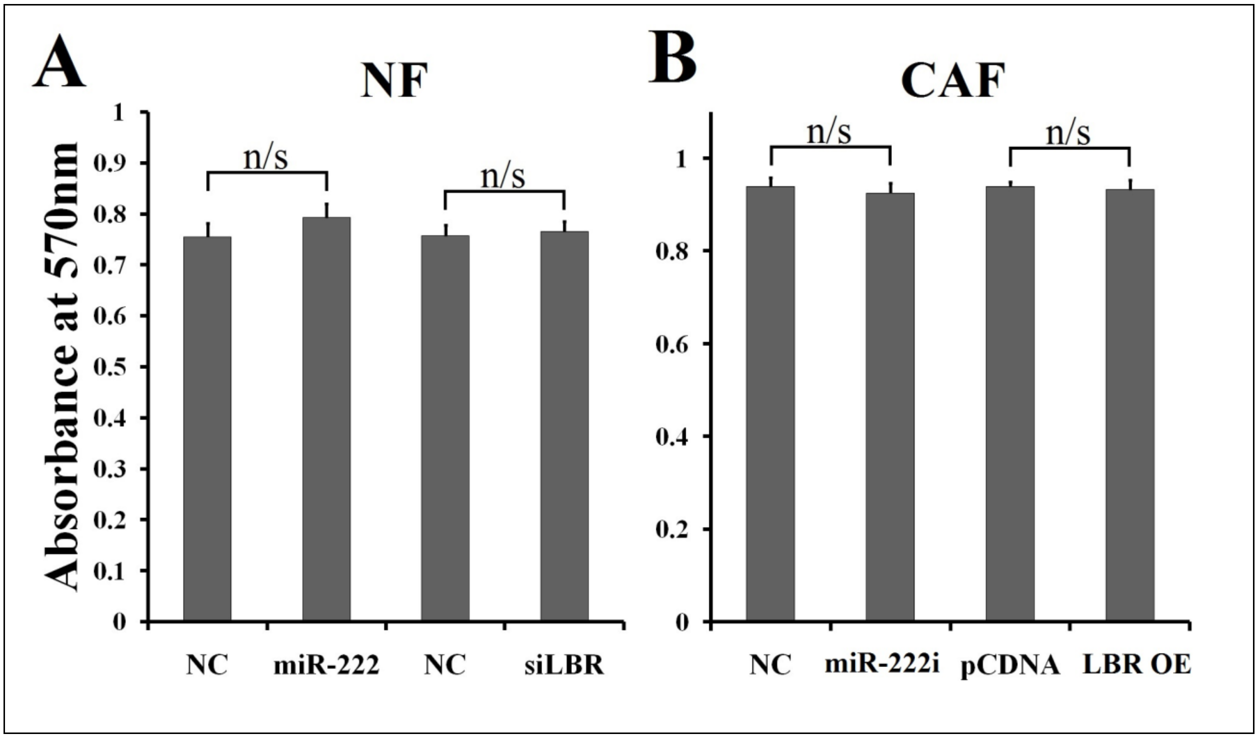

Supplementary Figure S3: Immortalized breast NFs (A) were transfected with miR-222 mimic or siRNA targeting LBR (siLBR) or appropriate controls (NC), and immortalized breast CAFs (B) were transfected with miR-222 inhibitor (i) or to over-express LBR (LBR OE) or with appropriate controls (NC, pCDNA). MTT assay were performed 72h post-transfection. Data represent biological triplicates.

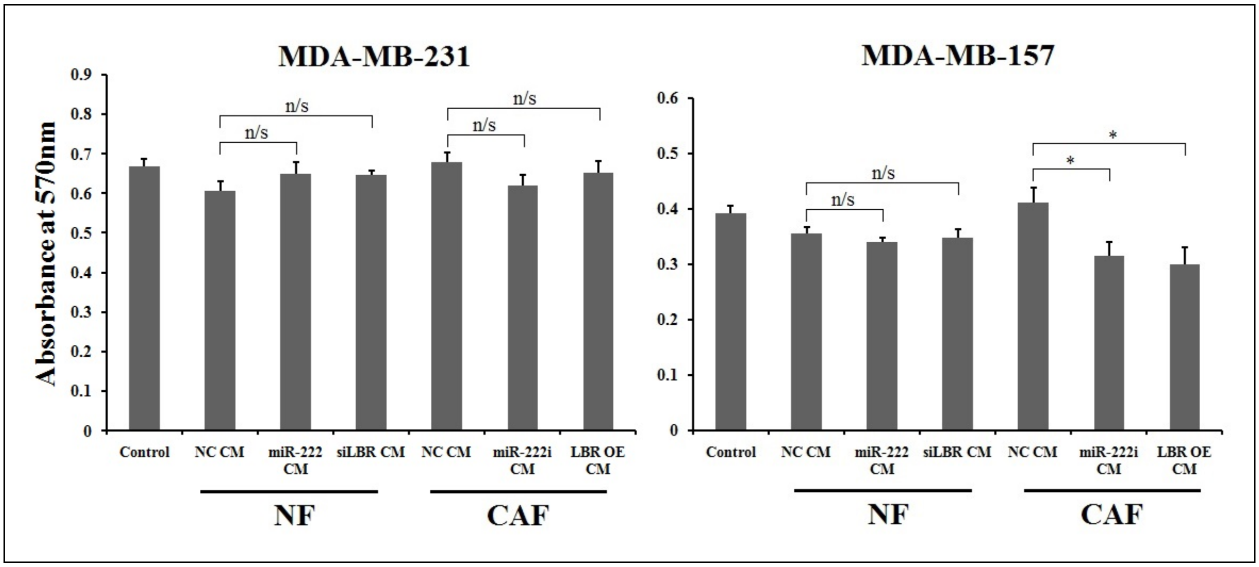

Supplementary Figure S4: Immortalized breast NFs were transfected with miR-222 mimic or siRNA targeting LBR (siLBR) or appropriate controls (NC), and immortalized breast CAFs were transfected with miR-222 inhibitor (i) or to over-express LBR (LBR OE) or with appropriate controls (NC).Conditioned medium (CM) was collected from fibroblast cultures and used to treat breast epithelial cancer lines MDA-MB-231 or MDA-MB-157. After that cells were seeded for the proliferation assay. Proliferation of MDA-MB-231(Left panel) or MDA-MB-157(Right panel) cell lines cultured with conditioned medium (CM) from the transfected fibroblasts as labelled was determined using MTT assay. Data represent biological triplicates, $p < 0.05$(*).

SUPPLEMENTARY TABLE S1(List of Primers)

GENE	FORWARD PRIMER	REVERSE PRIMER
ACTA2	GATGGTGGGAATGGGACAAA	GCCATGTTCTATCGGGTACTTC
18S	GTAACCCGTTGAACCCCATT	CCATCCAATCGGTAGTAGCG
IL6	ACCTTCCAAAGATGGCTGAA	CTGGCTTGTTCCTCACTACTC
IL8	GGAACCATCTCACTGTGTGTAA	GGGTGGAAAGGTTTGGAGTAT
CCL2	AGCAGCAAGTGTCCCAAA	TGTGGAGTGAGTGTTCAAGTC
FSP	TGGATGTGATGGTGTCCACCTT	CTGGAAAGCAGCTTCATCTGTC
VEGF	CAGGACATTGCTGTGCTTTG	CTCAGAAGCAGGTGAGAGTAAG
OPN	CATATGATGGCCGAGGTGATAG	AGGTGATGTCCTCGTCTGTA
MMP3	GTGAGGACACCAGCATGAA	GACCACTGTCCTTTCTCCTAAC
Vimentin	CAGCTTTCAAGTGCCTTTCTG	CTTGTAGGAGTGTCGGTTGTT
Slug	AACTACAGCGAACTGGACAC	GAGGATCTCTGGTTGTGGTATG
LBR	CTGGCAGTGAGAACCTTTGA	CAGGCCAAACATGATGAGAAAC
LBR Cloning	GTGGTACCACCATGCCAAGTAGGAA ATTTG	GCTCTAGAGCTTAGTAGATGTATGGAAAT ATACGG
LBR WT (Luciferase)	AAACTAGCGGCCGCTAGTAGTTGAA AATAAAATGTAGCAT	CTAGATGCTACATTTTATTTTCAACTACT AGCGGCCGCTAGTTT
LBR Mutated (Luciferase)	AAACTAGCGGCCGCTAGTAGTTGAA AATAAAATAGCGCAT	CTAGATGCGCTATTTTATTTTCAACTACT AGCGGCCGCTAGTTT
miR-222	AGCTACATCTGGCTACTGG	
